# Adenosine A_2A_ Receptors Control the Mis‐Localization of Aquaporin‐4 in Rats Subject to Repeated Restraint Stress

**DOI:** 10.1111/jnc.70450

**Published:** 2026-04-27

**Authors:** Liliana Dias, Samira G. Ferreira, Ana Margarida Nabais, Joana Silva, Rodrigo A. Cunha, Paula Agostinho

**Affiliations:** ^1^ CiBB ‐ Centre for Innovative Biomedicine and Biotechnology University of Coimbra Coimbra Portugal; ^2^ Department of Life Sciences, Faculty of Sciences and Technology University of Coimbra Coimbra Portugal; ^3^ Faculty of Medicine University of Coimbra Coimbra Portugal; ^4^ MIA‐Portugal, Multidisciplinary Institute of Aging University of Coimbra Coimbra Portugal

**Keywords:** adenosine A_2A_ receptor, anxiety, aquaporin‐4, astrocyte, gliosomes, stress

## Abstract

Repeated stress triggers anxiety accompanied by a deregulation of cortical glucocorticoid receptors, which is relieved by blocking adenosine A_2A_ receptors (A_2A_R). A_2A_R also controls the glymphatic system and the polarization of its key driver aquaporin‐4 (AQP4). Since the glymphatic system is altered upon repeated stress, we now tested if A_2A_R blockade could alleviate AQP4 polarization in rats subject to repeated restraint stress (RRS). As expected, RRS enhanced anxiety in the elevated plus maze, decreased self‐care behavior in the splash test, and decreased cortical glucocorticoid receptors levels; these alterations were prevented by daily treatment with the selective A_2A_R antagonist KW‐6002 (3 mg/kg/day). KW‐6002 treatment also prevented the RRS‐induced decrease of AQP4 density in gliosomes, corresponding to astrocytic membrane endfeet, the perivascular AQP4 polarization in astrocytes, and both perivascular and cellular AQP4 coverage. These findings show that A_2A_R controls AQP4 polarization upon restraint stress and prompt considering that A_2A_R might control the impact of repeated stress on brain dysfunction through a control of the glymphatic system.

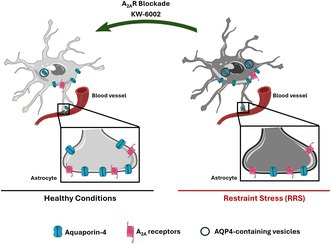

## Graphical abstract

Restraint stress triggers anxiety, along with a loss of polarization and coverage of aquaporin‐4 (AQP4) in astrocytes of the frontal cortex; these effects are attenuated by blockade of adenosine A_2A_ receptors.

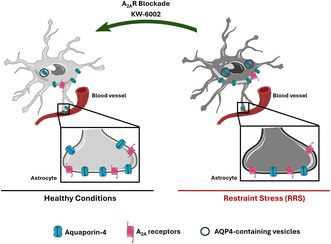

**Abbreviations**
A_2A_R: adenosine A_2A_ receptors
AQP4: aquaporin‐4
CTRL: control
EPM: elevated plus maze
FC: frontal cortex
GR: glucocorticoid receptors
ROI: regions of interest
RRID: research resource identifiers
RRS: repeated restraint stress

## 1 Introduction

Adenosine is a paracrine signal contributing to maintain homeostasis in different tissues, namely in the brain (IJzerman et al. 2022). Adenosine is released in an activity‐dependent manner and in particularly larger amounts under stressful conditions (Cunha 2016). This leads to an overfunction of adenosine A_2A_ receptors (A_2A_R) that contributes to mood dysfunction in different animal models subject to repeated stress conditions (Kaster et al. 2015; Wang et al. 2023). Thus, A_2A_R antagonists emerge as potential novel antidepressants, in accordance with the association of A_2A_R polymorphisms with depression (Oliveira et al. 2019) and with the inverse association between the intake of caffeine (a non‐selective adenosine antagonist) and the incidence of depression in different cohorts (Grosso et al. 2016; Unsal and Sanlier 2025).

Since A_2A_R control synaptic plasticity both at excitatory (e.g., Costenla et al. 2011) and inhibitory synapses (e.g., Kerkhofs et al. 2018), the ability of A_2A_R to control mood and depression has been conceived as resulting from a direct ability to rebalance neuronal activity (Cunha 2016). However, A_2A_R are also present in astrocytes and control numerous astrocytic functions (Paiva et al. 2019), such as glutamate uptake (e.g., Matos et al. 2012) or connexin‐43 activity (Madeira, Domingues, et al. 2023). The selective manipulation of astrocytic A_2A_R affects behavioral outputs such as memory performance (Matos et al. 2015; Orr et al. 2015) and modulates synaptic plasticity (Madeira, Lopes, et al. 2023; Dang et al. 2024), which alteration accompanies mood behavioral deficits in depressive conditions (Duman and Aghajanian 2012). In parallel, A_2A_R control other core processes involved in maladaptive stress such as the function of brain glucocorticoid receptor that orchestrate HPA axis dysfunction following repeated stress (Batalha et al. 2016). A_2A_R also control the glymphatic system (Sun et al. 2024), an aquaporin‐4 (AQP4)‐facilitated flow of cerebrospinal fluid through the brain parenchyma ensuring the clearance of metabolic wastes and protein deposits (Rasmussen et al. 2018), the dysfunction of which is linked to mood deterioration (Gu et al. 2022; Wen et al. 2024). In particular, A_2A_R control the polarization of AQP4 in astrocytes (Zhao et al. 2017; Sun et al. 2024; Dias et al. 2025), which is paramount to drive the glymphatic system (Mestre et al. 2018; Gomolka et al. 2023). Moreover, A_2A_R control alterations in cultured astrocytes triggered by the glucocorticoid analogue—dexamethasone (Madeira et al. 2022) and the loss of AQP4 polarization upon exposure to glucocorticoids (Dias et al. 2025). However, it is not known if the beneficial mood normalizing effect resulting from A_2A_R blockade is accompanied by an ability of A_2A_R blockade to prevent the mis‐localization of AQP4 occurring upon repeated stress (Bollinger et al. 2025). The relevance of AQP4 modulation was recently highlighted by reports showing that targeting AQP4 effectively reduces cerebral edema during the early acute phase in stroke preclinical models (Kitchen et al. 2020; Sylvain et al. 2021). Thus, we now tested if upon repeated restraint stress A_2A_R blockade affects AQP4 polarization. We focused on the prefrontal cortex, with a complementary analysis of hippocampus, due to the central role of these brain regions in the stress response and in mood and anxiety regulation (McEwen et al. 2016), as well as evidence that astrocytic AQP4 can be affected by maladaptive stress in these regions (Xia et al. 2017). The analyses were restricted to the glial fractions, considering that AQP4 is a protein predominantly expressed in astrocytes and therefore synaptic fractions are not expected to provide significant information regarding perivascular AQP4 localization. In addition, previous work demonstrated that astrocytic A_2A_R modulate AQP4 in astrocytes (Dias et al. 2025), supporting that glial fraction is the most relevant to address our hypothesis.

## 2 Materials and Methods

### 2.1 Animals

Adult male Wistar Han rats (8–12 weeks old), in a total number of 43 animals, were obtained from Charles River (Barcelona, Spain) and were maintained in groups of 2 from the same experimental group in the same cage. All statistical analyses were performed using an individual animal as the experimental unit (*n* refers to number of animals). The animals, with an initial weight of 260.30 ± 15.39 g, were arbitrarily assigned into four experimental groups: rats submitted to a repeated restraint stress (RRS) protocol or not (stress vs. control) with each group further sub‐divided into rats treated with a vehicle solution or with the selective adenosine A_2A_ receptor antagonist KW‐6002 (vehicle vs. KW‐6002). Accordingly, the distribution of the number of animals per group was as follows: control + vehicle (*n* = 14), control + KW‐6002 (*n* = 9), stress + vehicle (*n* = 12) and stress + KW‐6002 (*n* = 8). All animals were handled according to the principles and procedures outlined as “3R's” in the FELASA, ARRIVE, and European Union guidelines (86/609/EEC) and were approved by the Animal Welfare Committee of our Center (ORBEA_238_2019/1410/2019). The rats were maintained under a controlled environment (23°C ± 2°C, 12 h light/dark cycle, *ad libitum* access to food and water). All efforts were made to minimize animal suffering and to reduce the number of animals used. The sample size in the different experimental groups was calculated based on our previous experience using this model as well as the variability and range of expected effects in the behavioral and neurochemical analyses (Cognato et al. 2010; Kaster et al. 2015; Dias et al. 2021). Animals were coded using numbers and all analyses were performed in a blind manner.

### 2.2 Repeated Restraint Stress Protocol

We selected the repeated restraint stress (RRS) model, which we have previously shown to induce anxiety‐like behavior, as well as cognitive deficits, in adult male Wistar rats (Dias et al. 2021). This stress model is based on imposed physical immobility, which is a purely psychological stressor, a type highly prevalent in humans, and does not involve pain or physical exhaustion of the animals (Hammen 2005; Buynitsky and Mostofsky 2009).

The RRS model was carried out as done previously (Cunha et al. 2006; Dias et al. 2021). In a room adjacent to their colony, rats were placed individually in a DecapiCone immobilization bag (Braintree Scientific; cat. no. DC200) for 4 h every day during 14 consecutive days (between 9 a.m. and 1 p.m.). This protocol has previously been defined to be required to induce stable behavioral modifications for at least 1 week in adult male rats (Cunha et al. 2006). Each bag was secured around the tail of the rat and has a 1 cm hole at one end for breathing. After each daily restraint stress session, rats were returned to their home cages. Control group rats (weight‐ and age‐matched) were similarly handled, except that they were not immobilized; they also did not have access to food and water during the same period (4 h) in which stressed animals were under immobilization. All animals were weighed three times a week to assess weight gain, which is also a parameter used to estimate anxiety and, consequently, evaluate the success of the RRS protocol.

### 2.3 Chronic Treatment With KW‐6002

Previous studies have demonstrated the beneficial effects of adenosine A_2A_ receptors (A_2A_R) blockade on mood and memory dysfunctions, including through KW‐6002 administration (e.g., Cognato et al. 2010; Kaster et al. 2015). KW‐6002, also designated as istradefylline [8‐[(1E)‐2‐(3,4‐dimethoxyphenyl)ethenyl]‐1,3‐diethyl‐3,7‐dihydro‐7‐methyl‐1H‐purine‐2,6‐dione; Tocris, cat. no. 5147], is a selective A_2A_R antagonist and an approved drug for Parkinson's disease adjunct treatment (Chen and Cunha 2020). A dose of KW‐6002 of 3 mg/kg/day was selected according to previous testing of its efficacy and selectivity after oral administration, having a good to moderate bioavailability (61% for oral administration), high half‐life and permeability through the blood–brain barrier and reaching sufficient brain levels (Aoyama et al. 2002; Yang et al. 2007). Administration via the drinking water was previously used by our group (Cognato et al. 2010; Kaster et al. 2015) and was selected since it is a non‐invasive method suitable for prolonged drug delivery, minimizing additional handling and procedural stress that could interfere with the RRS paradigm. Although this approach does not allow complete control of individual drug intake, water consumption was monitored throughout the protocol and no appreciable differences were detected between experimental groups.

KW‐6002 was diluted in drinking water with 0.5% methylcellulose at an amount calculated to achieve the dose of 3 mg/kg/day (Cognato et al. 2010; Kaster et al. 2015). Briefly, the daily amount of KW‐6002 required per rat was first estimated considering the average body weight of the animal and this value was then used to determine the appropriate concentration in the drinking water based on the mean daily water consumption (approximately 35 mL per rat). The administration of KW‐6002 was initiated 3 days before the onset of the RRS protocol, continued throughout the RRS protocol and until the sacrifice of the animals. All analyses were performed *post hoc* at the end of the experimental period. The KW‐6002 solution was placed in light‐protected bottles, being continuously available. For the control group, a vehicle solution containing 0.5% methylcellulose diluted in the drinking water was given during the same period. Rats housed in the same cage were age‐ and weight‐matched, shared a single drinking bottle and were always assigned to the same treatment condition (vehicle or KW‐6002).

### 2.4 Behavioral Evaluation

The behavioral tests were performed between 9 a.m. and 5 p.m., under dim red light (3–12 lx), starting in the day after the end of the RRS protocol. Prior to the start of behavioral tests, rats were transferred to the experimental room, allowing habituation to the new environment for at least 1 h. The analyses were performed using an automated tracking system based on video feed (ANY‐maze v.6 software, Stoelting, Ireland; paired with a Logitech webcam), and only the splash test was analyzed manually. After each trial, the apparatus was cleaned with 10% ethanol solution and dried with paper wipes to remove and disperse olfactory, tactile and visual clues.

### 2.5 Elevated Plus Maze

The elevated plus maze (EPM) is commonly used to evaluate anxiety‐like behavior in rodents, and it is based on their natural curiosity to explore new environments, as well as on their innate fear of open spaces (Montgomery 1955). The apparatus consists of four arms of the same size (40 cm × 5 cm), with two closed arms perpendicular to two open arms, arranged in the form of a cross, and elevated 50 cm from the ground. Two opposed arms were surrounded by 30 cm high opaque black Plexiglas walls, except for the entrance (closed arms), while the other two arms had no walls (open arms). The animals were tested in the EPM 1 day after the final restraint/control handling session. Each rat was placed in the center of the four arms connection, facing the open arm opposite the experimenter; therefore, the animals were confronted with an avoidance‐approach conflict, in which they had to choose between staying protected in the enclosed arms or investigating novel, but unprotected open arms (Rodgers and Dalvi 1997). The behavior of the animals was recorded for 5 min and analyzed using the video‐tracking software (Any‐Maze). We measured the number of entries and the time spent in both open and closed arms, considering an entry only when the whole body and the four paws were inside an arm. Thus, the level of anxiety was inversely proportional to the time spent in the open arms. Additionally, the traveled distance was used as an indicator of locomotor activity.

### 2.6 Splash Test

Grooming behavior has been used as an indicator of self‐care and motivation, being associated with anxiety: short time of grooming behavior indicates high anxiety levels in stressed rodents (Willner 2005; Isingrini et al. 2010). To evaluate grooming, rats were sprayed with a 10% sucrose solution diluted in water on the dorsal coat surface and then placed alone in a Plexiglas arena (30 × 16 × 19 cm) for 5 min. Grooming behavior was assessed by measuring the time spent licking, biting, or scratching the fur to clean the solution and the latency for the first grooming event, both expressed in seconds.

### 2.7 Total Protein Extracts

Total protein extracts correspond to a tissue homogenate that includes the whole protein content, including cytoplasmic, nuclear and membrane proteins as well as proteins from all cellular compartments and cell types. This allows evaluating the total protein content in a specific region, in this case the frontal cortex or the hippocampus. Before collecting these brain regions, the rats used in behavioral studies were deeply anesthetized by inhalation of halothane (2‐bromo‐2‐chloro‐1,1,1‐trifluoroethane, Sigma‐Aldrich cat. no. B4388), being placed in an airtight chamber containing a halothane‐saturated atmosphere and, once unconscious, were sacrificed by decapitation, following the procedure previously used by our group (Dias et al. 2021; Simões et al. 2023) and others (Serpa et al. 2009). The brain was quickly removed, and the brain regions were dissected at 4°C. Afterwards, the tissue (5–10 mg) was homogenized in RIPA buffer (150 mM NaCl, 1% IGEPAL CA‐630; Sigma‐Aldrich cat. no. I8896), 0.5% sodium deoxycholate, 0.1% sodium dodecyl sulfate (SDS) and 50 mM Tris (pH 8.0) supplemented with 0.001% protease inhibitor cocktail (CLAP, Merck, cat. no. 04693116001), 1 mM dithiotreitol (DTT, Merck, cat. no. 43816) and 1 mM phenylmethylsulfonyl fluoride (PMSF, Merck, cat. no 52332), using a glass‐Teflon tissue grinder. Homogenates were agitated for 2 h at 4°C, the samples were centrifuged at 13 000×*g* for 20 min at 4°C and then the supernatant was collected and stored at −20°C for further analysis by Western blot.

### 2.8 Preparation of Gliosomes

We have previously used and characterized gliosomes, which correspond to vesicles of membranes from astrocyte terminals or endfeet (Matos et al. 2012). The preparations were obtained from brain tissue using a discontinuous Percoll gradient, as previously described (Matos et al. 2012). Briefly, after deep halothane anesthesia of the animals followed by decapitation, the brain was quickly removed and the frontal cortex and hippocampus were rapidly dissected. Each tissue was homogenized in a sucrose solution (0.25 M sucrose, 10 mM HEPES, pH 7.4 at 4°C) using a glass‐Teflon tissue grinder (clearance 0.15 mm). The homogenate was centrifuged (5 min, 1000×*g* at 4°C) to remove nuclei and cell debris. The supernatant was placed on the top of a discontinuous gradient composed of 2%, 6%, 10%, and 23% v/v Percoll (Sigma‐Aldrich, cat. no. GE17‐0891‐02) in a sucrose solution (0.32 M sucrose and 1 mM EDTA, pH 7.4 at 4°C), which was then gently stratified by centrifugation at 31 000×*g* for 5 min, with braking speed turned off after reaching 2000×*g* to avoid a sudden stop and the consequent gradient disruption. Gliosomes were collected in the interface between the 2% and 6% v/v Percoll layers, washed in 8 mL of isotonic physiological solution (140 mM NaCl, 5 mM KCl, 5 mM NaHCO_3_, 1.2 mM NaH_2_PO_4_, 1 mM MgCl_2_, 10 mM glucose and 10 mM HEPES, pH 7.4 at 4°C) and further centrifuged at 30 000×*g* for 20 min at 4°C. Pellets were washed again in isotonic physiological solution and centrifuged at 16 000×*g* for 20 min at 4°C. The pellets were re‐suspended in RIPA buffer (150 mM NaCl, 1% IGEPAL, 0.5% sodium deoxycholate, 0.1% SDS, and 50 mM Tris, pH 8.0) supplemented with 0.001% CLAP, 1 mM DTT, 1 mM PMSF, and stored at −20°C for further analysis by Western blot.

### 2.9 Western Blot

After obtaining the total extracts and the gliosomes fraction, the amount of protein was determined using the bicinchoninic acid method (BCA, Thermo Fisher Scientific, cat. no. 23250). Samples were then denaturated by heating at 70°C for 20 min, following the addition of 6× concentrated sample buffer (500 mM Tris, 600 mM DTT, 10.3% sodium dodecyl sulfate, 30% glycerol and 0.012% bromophenol). After loading the samples (20 μg for AQP4 and 10 μg for glucocorticoid receptors), the proteins were electrophoretically separated in a 10% polyacrylamide SDS‐PAGE resolving gel and a 4% polyacrylamide SDS‐PAGE stacking gel in running buffer (25 mM Tris, 192 mM bicine, 0.1% SDS, pH 8.3). Proteins were then wet electro‐transferred to nitrocellulose membranes (GE Healthcare, cat. no. 17‐0891‐01) at 1 A for 2 h in a CAPS solution (10 mM CAPS, pH 11.0, 10% methanol). Transfer efficacy and equal protein loading were verified by a reversible staining with Ponceau S solution (Merck, cat. no. P7170) for 5 min and then the membranes were washed with water. Nonspecific binding was blocked with 5% non‐fat dry milk in Tris‐buffered saline (TBS: 20 mM Tris, 137 mM NaCl, pH 7.6) containing 0.1% Tween‐20 (TBS‐T) for 1 h at room temperature. The membranes were further incubated overnight at 4°C with the primary antibodies diluted in 1% non‐fat milk in TBS‐T: guinea‐pig anti‐AQP4 (1:1500, Synaptic Systems, RRID: AB_2802156) or rabbit anti‐glucocorticoid receptors (1:1500, Santa Cruz Biotechnology, RRID:AB_2155786). After rinsing with TBS‐T, the membranes were incubated with the appropriate peroxidase conjugated goat anti‐guinea pig (1:5000, Thermo Fisher Scientific, RRID: AB_258247) or anti‐rabbit IgG secondary antibodies (1:5000, Thermo Fisher Scientific, RRID: AB_228338) for 2 h at room temperature. Membranes were revealed with enhanced chemiluminescence substrate Pierce ECL Western Blotting Substrate (Thermo Fischer Scientific, cat. no. 32106) and visualized using an imaging system (Chemidoc, RRID: SCR_021693). After immersing twice for 15 min in stripping solution (200 mM glycine; 0.1% SDS and 1% Tween 20, pH 2.2) to remove primary and secondary antibodies, the membranes were reprobed for β‐actin (1:20000, Sigma Aldrich, RRID: AB_47674) and peroxidase‐conjugated goat anti‐mouse IgG secondary antibody (1:5000, Thermo Fisher Scientific, RRID: AB_228302) to control for protein loading. In the case of GR immunolabeling, a test was performed using frontal cortex samples from both mice and rats to validate the use of this antibody to detect GR in rat tissue. The densiometric analysis of protein bands was performed using Image Lab Software with the version 5.2.1 (Bio‐Rad, RRID: SCR_014210).

### 2.10 Preparation of Brain Sections

Rats were anesthetized using a freshly prepared solution of avertin 20 mg/mL (70.7 mM 2,2,2‐tribromoethanol (TBE; Sigma‐Aldrich, cat. no. T48402), 450 mM 2‐methyl‐2‐butanol (Merck, cat. no. 19954) in a phosphate buffered solution, PBS: 135 mM NaCl, 2.7 mM KCl, 4.3 mM Na_2_HPO_4_, 1.47 mM KH_2_PO_4_, pH 7.4), which was filtered through a 0.2 μm sterilize filter. Particular care was taken to prevent the degradation of the avertin solution by protecting it from light, keeping it at 4°C, being fresh solution prepared for each batch of animals and used within 1 week. The avertin solution was administered at a dose of 12 μL/g of rat body weight by intraperitoneal injection. This non‐volatile anesthesia has been used by our group over the years as it provides rapid induction and reliable depth of anesthesia with a single use, and is appropriate for terminal procedures, such as stereotaxic surgeries and transcardiac perfusion (e.g., Matos et al. 2012; Lopes et al. 2022), with a very low morbidity or lethality (reviewed Meyer and Fish 2005). The use of avertin anesthesia was approved by the controlling official entities (ORBEA and DGAV). After confirming the state of deep anesthesia of rats (no reaction to paw and tail pinch), the beating heart of the anesthetized animal was exposed, the descending aorta was clamped, and a catheter was inserted in the ascending aorta through the left ventricle and the right atrium was opened to allow the outflow of perfusate. The animal was then perfused with 200 mL cold phosphate buffered saline (PBS: 135 mM NaCl, 2.7 mM KCl, 4.3 mM Na_2_HPO_4_, 1.47 mM KH_2_PO_4_), followed by 200 mL of 4% paraformaldehyde in saline solution. Then, the brains were removed and kept 24 h in the same paraformaldehyde solution at 4°C. Subsequently, the brains were transferred to a solution of 30% sucrose in PBS at 4°C until reaching the bottom of the solution (at least 48 h later), being further cryopreserved by fast freezing in Cytocool (Thermo Fisher Scientific, cat. no. 8323) and stored at −80°C until sectioning, as previously described with modifications (Madeira, Lopes, et al. 2023). The brains were then embedded in Tissue‐Tek Optimal Cutting Temperature matrix (Sakura‐Americas, cat. no. 4583), frozen at −20°C and sectioned in 30 μm‐thick coronal sections using a cryostat (CM3050S from Leica Microsystems, Germany). The sections were stored in anti‐freezing solution (12.3 mM NaH_2_PO_4_, 20.3 mM NaHPO_4_, 30% v/v ethylene glycol, 30% v/v glycerol) and the subsequent immunostaining was performed in free floating slices.

### 2.11 Immunohistochemistry

Coronal sections containing the medial prefrontal cortex were washed 3 times for 5 min in PBS at room temperature and then permeabilized with 0.1% Triton‐X100 in PBS for 15 min. Brain sections were then submerged in blocking solution with 5% horse serum in 0.1% Triton‐X100 solution for 2 h. The sections were subsequently incubated with the primary antibody guinea‐pig anti‐AQP4 (1:600, Synaptic Systems, RRID:AB_2802156) in blocking solution for 24 h at 4°C under agitation. In the following day, the sections were washed 3 times for 10 min with PBS containing 0.1% Triton‐X100 and incubated with the secondary antibody (goat anti‐guinea pig conjugated with Alexa‐fluor 594, Thermo Fisher Scientific, RRID: AB_2534120) for 2 h at room temperature in blocking solution. When evaluating aquaporin‐4 immunofluorescence, sections were concomitantly incubated with 10 μg/mL *Lycopersicon esculentum* lectin DyLight 488 (Invitrogen, cat. no. L32470) to label blood vessels. After washing 3 times for 10 min, the sections were incubated in 4′,6‐diamidine‐2′‐phenylindole dihydrochloride (DAPI, 1:5000) for 10 min for nuclear staining. The sections were washed 3 times with PBS and mounted in gelatine‐coated slides with Fluoromount mounting medium (Sigma). Immunohistochemical controls were taken by omitting the primary antibodies and replacing them with blocking solution. Sections were observed and images were acquired in a LSM 710 confocal microscope (Carl Zeiss) with a Plan‐Apochromat oil‐immersion 40× or 63× objectives and excited using and Argon/2, DPSS 561–10 and Diode 405–30 lasers at 488 nm, 561 nm and 405 nm, respectively. Emission was recorded using a QUASAR multichannel photomultiplier detector (Zeiss) and data acquisition were controlled by the Zen Black software. Analyses were performed using ImageJ software (NIH, Bethesda, USA).

### 2.12 Analysis of Aquaporin‐4 (AQP4) Immunofluorescence

The localization of AQP4 was investigated using images obtained with LSM 710 confocal microscope (Carl Zeiss) with a Plan‐Apochromat oil‐immersion 40× objective after immunohistochemistry labelling, as described above. Composite micrographs were separated into their respective channels, the lectin channel (used to label blood vessels) was duplicated and thresholded to generate a binary mask identifying lectin‐positive vascular structures. This mask was used to define regions of interest (ROIs) corresponding to blood vessels. Next, to isolate AQP4 signal associated with vascular endfeet, these ROIs were then applied to the original AQP4 fluorescence images to measure mean unthresholded AQP4 fluorescence intensity and area within vasculature‐associated regions, which we refer to as perivascular AQP4 signal. To obtain the non‐vascular cellular AQP4 signal, the perivascular AQP4 image was subtracted from the original AQP4 image, and the mean value and area was recorded. AQP4 polarization was expressed as the ratio between perivascular AQP4 immunofluorescence and mean fluorescence intensity of cellular AQP4. Area coverage was computed by dividing the ROI area of perivascular AQP4 by total image area. We analyzed the average of 5 images/ROI set per animal, obtained from 2 to 3 different sections of the prefrontal cortex.

### 2.13 Statistical Analysis

Data are presented as mean ± SEM (standard error of the mean) for imaging and biochemical experiments or mean ± SD (standard deviation) for behavioral experiments, of *n* experiments (number of different animals). For imaging quantifications, five fields were analyzed per animal and values were averaged to yield a single data point per animal, which was used as the unit for statistical analysis. The confidence interval was set as 95% so that the difference between means was considered significant at *p* values < 5% (*p* < 0.05). Data were first confirmed to be normally distributed, as verified using Shapiro–Wilk tests, before carrying out parametric analysis using one‐way ANOVA followed by Dunnett's *post hoc* test or two‐way ANOVA followed by Tukey multiple comparison *post hoc* test, considering the presence of one or two independent variables, respectively. Rat weight gain over time was evaluated using repeated measures (RM) two‐way ANOVA with Geisser–Greenhouse correction. This approach was used consistently across datasets to appropriately account for multiple group comparisons and control the familywise error rate. No formal exclusion criteria were pre‐established for the study. No animals were entirely excluded, and no animals were replaced. Animals were excluded from behavioral analyses when the animal did not move during the session, preventing reliable behavioral scoring. In addition, detection of outliers was performed using the ROUT analysis (*Q* = 1%) and the identified data points were excluded from analysis. All analyses were performed in a blind manner, including the data analysis. Data were analyzed using GraphPad Prism software, version 8.0 (RRID: SCR_002798).

## 3 Results

### 3.1 Effect of Repeated Restraint Stress (RRS) on Anxiety Behavior

**FIGURE 1:**
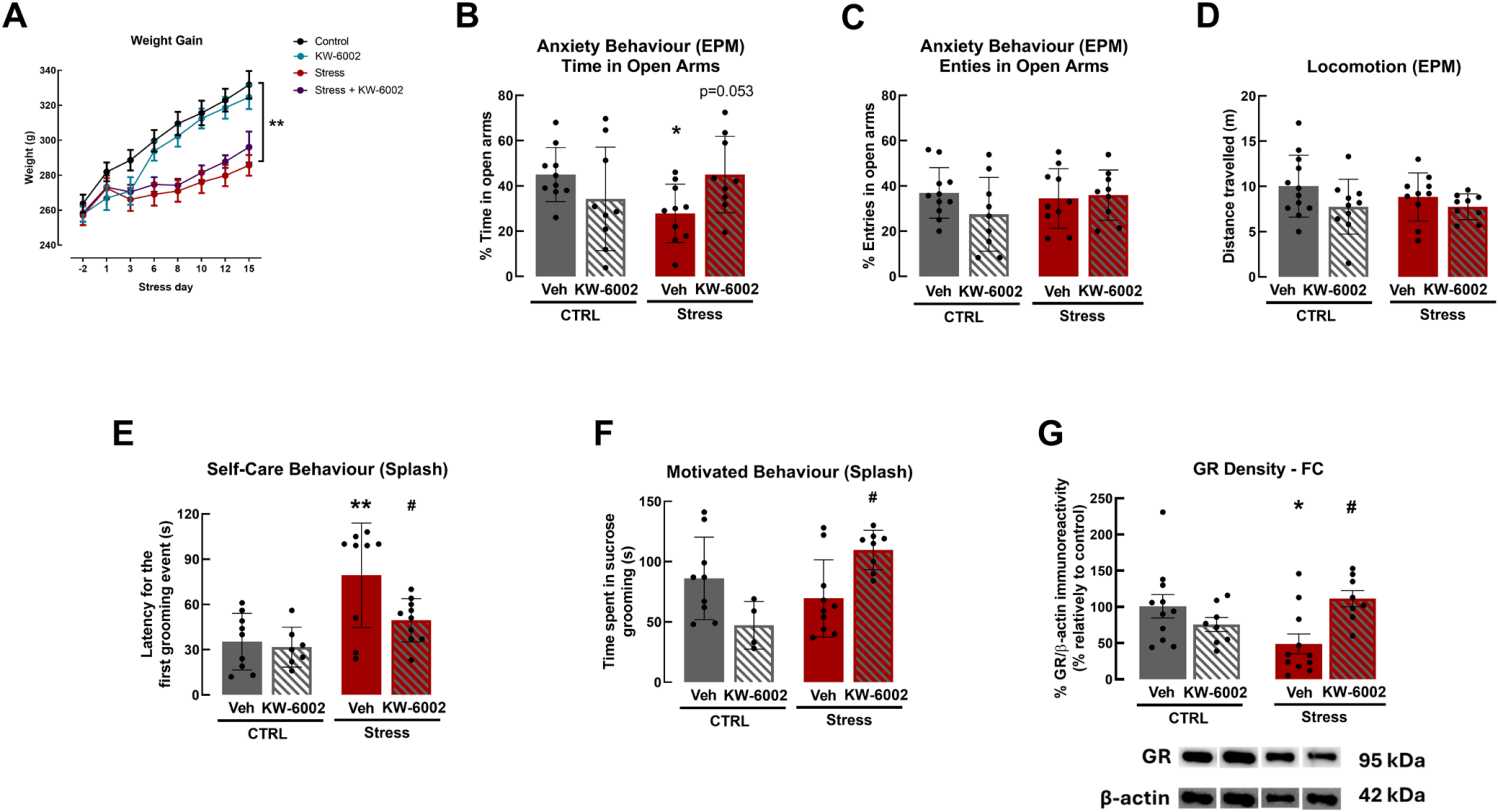
Impact of adenosine A_2A_ receptors (A_2A_R) on anxiety‐like behavior in Wistar rats (8 weeks old) submitted to repeated restraint stress (RRS). RRS decreased weight gain but A_2A_R blockade with KW‐6002 consumption through the drinking water (3 mg/kg/day) did not affect rat weight gain (A). KW‐6002 intake prevented the decreased time spent in open arms of an elevated plus maze induced by RRS (B), although no differences were observed either in the number of entries in the open arms (C) or in the locomotion (D). KW‐6002 intake decreased the latency for the first grooming event in the sucrose splash test (E) and increased total grooming time (F) in RRS rats. In RRS rats, KW‐6002 intake prevented the decrease in the density of glucocorticoid receptors in the frontal cortex (FC), restoring it to levels comparable to the control group (CTRL) (G). Representative images (rearranged for presentation purposes) of GR and β‐Actin (loading control protein) immunoblots are shown below the respective panel. Data are mean ± SD of 4–11 rats per group. *p < 0.05, **p < 0.01 compared with CTRL, using a one‐way ANOVA followed by Dunnett's *post hoc* test; #*p* < 0.05 compared with RRS animals, using a two‐way ANOVA, followed by *post hoc* Tukey's test. Animal weight gain over time was analyzed using repeated measures (RM) two‐way ANOVA with Geisser–Greenhouse correction.

The study of the impact of increased anxiety and maladaptive stress in brain function requires reliable models with validated phenotype. Hence, we first confirmed that RRS triggered an anxiety‐like behavior. Indeed, male Wistar Han rats submitted to the RRS protocol (Stress) showed a decreased weight gain over time as compared with control rats (CTRL), not submitted to RRS (CTRL: 331.8 ± 7.2 g; Stress: 285.7 ± 3.1 g; *F*_72,216_ = 2.46, *p* = 0.001, *n* = 10; Figure 1A). RRS significantly decreased by 40% the time spent by rats in the open arms of the elevated plus maze (CTRL: 45.02 ± 3.78 s; Stress: 27.86 ± 4.07 s; *F*_2,26_ = 2.81, *p* = 0.049, *n* = 10; Figure 1B), which indicates an anxiety‐like phenotype. However, no differences were observed either in the number of entries in the open arms or in locomotion of the animals (Figure 1C,D). Additionally, we investigated the impact of RRS in the self‐care of rats, assessed by the evaluation of animal grooming in the sucrose splash test. An increased latency of 44 s was observed for the first grooming event (CTRL: 35.33 ± 6.25 s, Stress: 79.33 ± 11.55 s; *F*_2,22_ = 9.82, *p* = 0.002; *n* = 9) and a small decrease in total grooming time in the animals submitted to RRS, indicating a decrease in self‐care behavior, as presented in Figure 1E,F. In a total homogenate of the frontal cortex, we also evaluated the density of glucocorticoid receptors, which have a pivotal role in stress response in this brain region (Arnsten 2009). The results show a decreased density of glucocorticoid receptors in the frontal cortex of RRS rats (CTRL: 100.80% ± 18.17%, Stress: 48.91% ± 13.72%; *F*_2,27_ = 3.65, *p* = 0.040; *n* = 11; Figure 1G). The hippocampus is also involved in anxiety regulation (McEwen et al. 2016); however, we did not observe significant differences in glucocorticoid receptors density in the hippocampus of RRS rats (*t*_17_ = 1.21, *p* = 0.245, *n* = 8–11, Figure S1A) and, therefore, subsequent analyses focused on the frontal cortex. These results indicate that the RRS protocol induced an anxiety‐like phenotype, paralleled by a decreased self‐care behavior and a reduction of glucocorticoid receptors density in the frontal cortex, constituting a reliable animal model for maladaptive stress.

### 3.2 Impact of RRS on Aquaporin‐4 Density and Polarization

**FIGURE 2:**
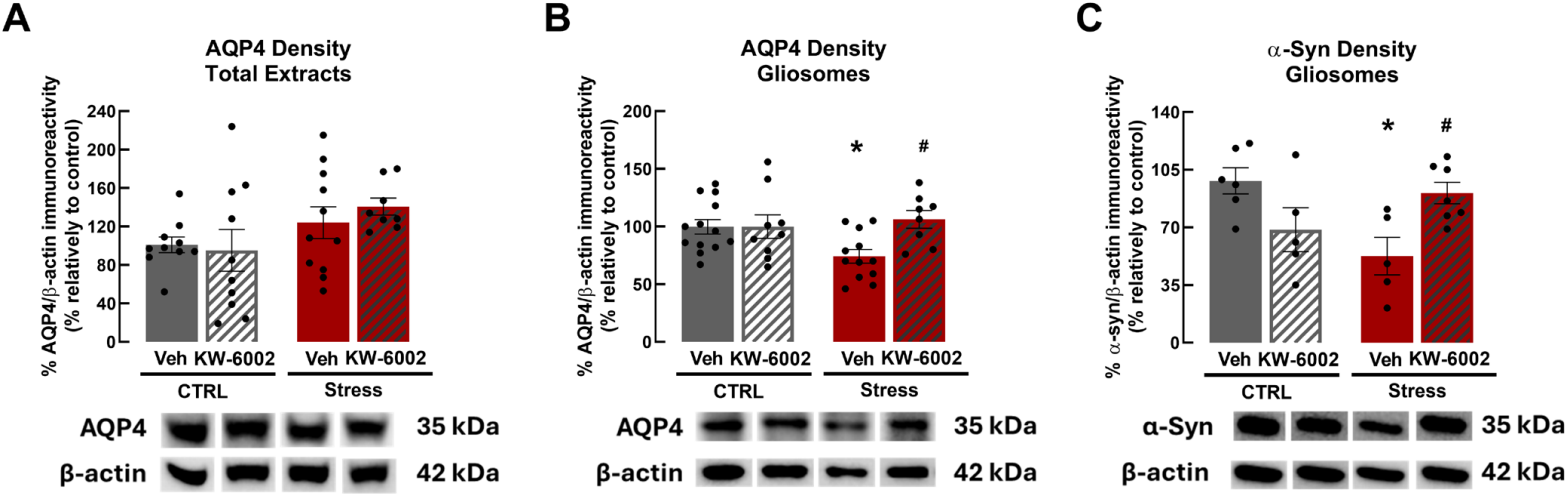
Repeated restraint stress (RRS) in male Wistar rats lowers AQP4 density at astrocytic endfeet, an effect prevented by A_2A_R blockade. In the frontal cortex (FC), RRS (Stress) did not affect total aquaporin‐4 (AQP4) density evaluated by Western blot (A). In FC gliosomes, corresponding to astrocytic membrane endfeet, RRS (Stress) significantly decreased AQP4 (B) and α‐syntrophin (C) densities, these effects being prevented by the A_2A_R antagonist KW‐6002. Representative images (rearranged for presentation purposes) of AQP4 and β‐Actin (loading control protein) immunoblots are shown below the respective panel. Data are mean ± SEM of 7–13 rats per group. * *p* < 0.05 as compared with non‐stressed animals (control, CTRL) using one‐way ANOVA followed by Dunnett's *post hoc* test; #*p* < 0.05 as compared with RRS animals using a two‐way ANOVA, *post hoc* Tukey's test.

To regulate fluid and solute exchanges, aquaporin‐4 (AQP4) is mainly located in astrocytes, particularly at endfeet in close proximity to blood vessels (Hoddevik et al. 2017). Alterations in AQP4 density, mainly in perivascular regions where it reflects AQP4 polarization, constitute an important factor in pathological conditions (e.g., Iliff et al. 2012; Zhao et al. 2017). First, we evaluated total AQP4 density in the frontal cortex of rats and did not observe differences between control and RRS groups in total extracts of the frontal cortex (*F*_3,35_ = 1.69, *p* = 0.577, Figure 2A). Considering the crucial role of AQP4 in astrocytic endfeet, we investigated the impact of RRS on AQP4 density particularly in gliosomes, which encompass vesicles of membranes of those endfeet. We found that RRS significantly decreased AQP4 density in gliosomes from the frontal cortex by approximately 25% (CTRL: 99.77% ± 6.27%, Stress: 74.11% ± 5.92%; *F*_2.31_ = 4.38, *p* = 0.024, *n* = 12–13, Figure 2B), indicating an AQP4 mis‐localization from astrocytic endfeet. This contention is reinforced by the observed decrease in the density of α‐syntrophin, a protein that is directly related to AQP4 localization in the membrane of astrocytic processes (Amiry‐Moghaddam et al. 2003; Camassa et al. 2015) (CTRL: 98.33% ± 13.80%, Stress: 52.60% ± 12.68%; *F*_2,13_ = 4.81, *p* = 0.018, *n* = 5–6, Figure 2C). Conversely, RRS did not significantly affect the density of AQP4 in total extracts or gliosomes from the hippocampus (*p* > 0.05, *n* = 9–14, Figure S1B,C), indicating that RRS did not impact hippocampal AQP4 density and location. Considering the absence of detectable AQP4 alterations in the hippocampus, subsequent experiments were performed only in the prefrontal cortex, where RRS‐induced alterations were evident and associated with behavioral alterations.

**FIGURE 3:**
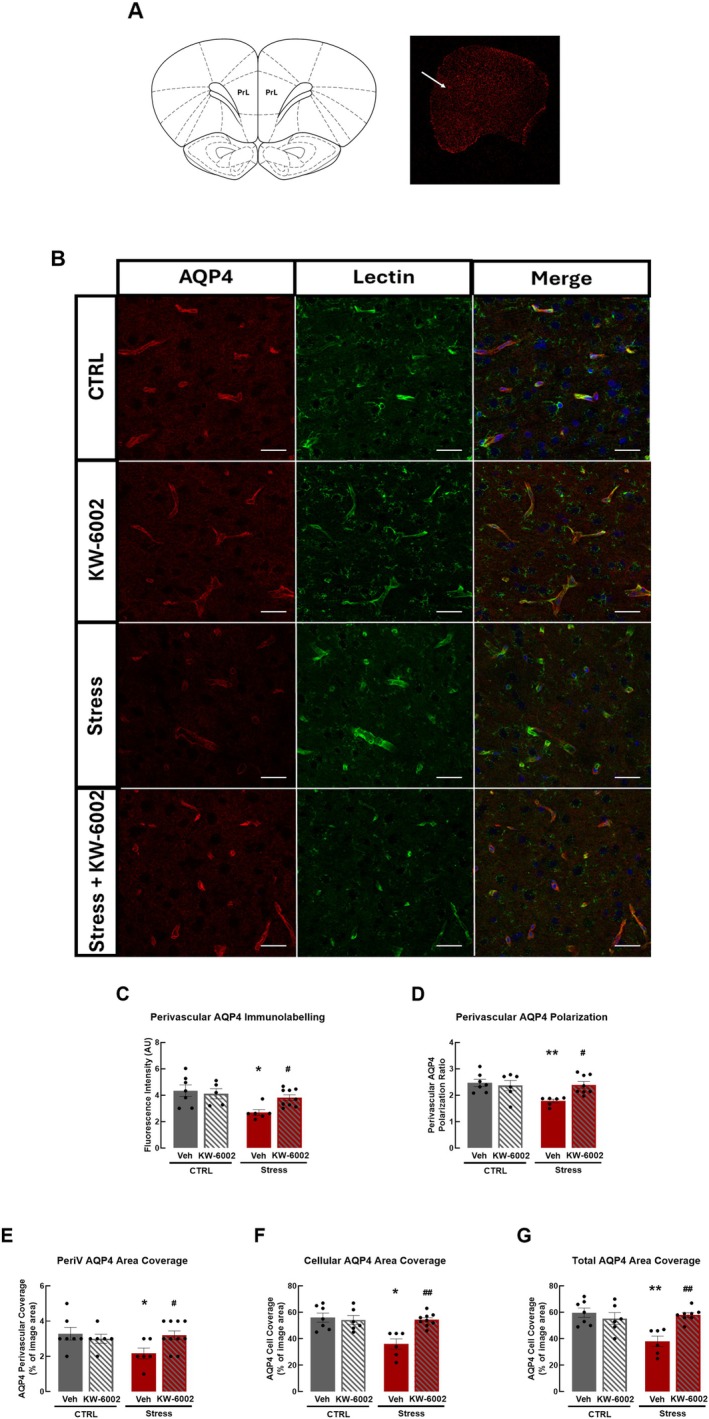
Impact of A_2A_R blockade on AQP4 polarization and coverage. Schematic representation of the brain region analyzed (prelimbic area, PrL), corresponding to the arrow in the representative (half of hemisphere) overview image (A). Representative images showing AQP4 proximity with blood vessels through lectin labelling (B) indicate that A_2A_R blockade prevented the repeated restraint stress (RRS)‐induced decrease in AQP4 immunolabeling in perivascular regions (C) and in perivascular AQP4 polarization (D). KW‐6002 prevented the RRS‐induced decrease in both perivascular AQP4 area coverage (E) and cellular AQP4 area coverage (F) and also increased total AQP4 area coverage (G). For each animal, quantification was performed across 4–5 fields and averaged to yield one value per animal. Data are mean ± SEM of 6–9 rats per group. *p < 0.05, **p < 0.01 as compared with non‐stressed animals (control, CTRL) using a one‐way ANOVA followed by Dunnett's *post hoc* test; #*p* < 0.05, ##*p* < 0.01 as compared with RRS animals using a two‐way ANOVA, *post hoc* Tukey's test. Scale bar represents 50 μm.

We observed in the frontal cortex that AQP4 is abundantly located in perivascular regions, as observed by its proximity to blood vessels, assessed through lectin labelling (Figure 3). This allowed us to evaluate AQP4 polarization in these regions. We found that RRS significantly decreased AQP4 immunolabeling in perivascular regions in the frontal cortex (CTRL: 4.35 ± 0.43 a.u., Stress: 2.69 ± 0.22 a.u.; *F*_2,15_ = 6.03, *p* = 0.009; *n* = 6–7; Figure 3C), leading to a decreased AQP4 polarization ratio in RSS animals as compared with non‐stressed controls (CTRL: 2.47 ± 0.14 a.u., Stress: 1.78 ± 0.07 a.u.; *F*_2,16_ = 6.70, *p* = 0.006; *n* = 6–7; Figure 3B,D). Furthermore, we evaluated the area covered by AQP4, designated as area coverage, in both perivascular and non‐perivascular regions, being the later designated as cellular coverage. We found that RRS decreased both perivascular area coverage (CTRL: 3.29% ± 0.36%, Stress: 2.17% ± 0.31%; *F*_2,16_ = 3.32, *p* = 0.043; *n* = 6–7; Figure 3E) and cellular area coverage (CTRL: 56.00% ± 3.42%, Stress: 36.00% ± 3.90%; *F*_2,16_ = 9.24, *p* = 0.002; *n* = 6–7; Figure 3F), which consequently decreased total area coverage (CTRL: 59.71% ± 3.50%, Stress: 38.00% ± 3.95%; *F*_2,16_ = 8.42, *p* = 0.002; *n* = 6–4; Figure 3G). Overall, these results showed that RRS impaired AQP4 density, polarization and area coverage, which might impact AQP4 function, being consistent with an impairment of the glymphatic system.

### 3.3 Adenosine A_2A_ Receptors (A_2A_R) Regulate Behavioral Alterations Induced by RRS

We have previously demonstrated that the blockade of A_2A_R has beneficial effects in animal models with impaired mood‐related behaviors (Kaster et al. 2015; Dias et al. 2021; Wang et al. 2023). We now first confirmed that A_2A_R blockade indeed prevented anxiety‐like behavior induced by RRS. Although the blockade of A_2A_R using the selective antagonist KW‐6002, administered via drinking water, did not affect weight gain or the locomotion of rats (Figure 1A,D), it prevented the anxiety phenotype, as observed by an increase in RRS rats of the time spent in the open arms of the elevated plus maze (Stress: 27.86 ± 4.07 s, Stress+KW‐6002: 48.23 ± 7.79 s; *F*_1,33_ = 8.61, *p* = 0.053; *n* = 8–10; Figure 1B), to levels similar to these of non‐stressed rats (CTRL: 45.02 ± 17.16 s, *F*_1,33_ = 8.61, *p* = 0.974, *n* = 8–10). Furthermore, A_2A_R blockade significantly decreased the latency for the first grooming event (Stress: 79.33 ± 11.55 s, Stress+KW‐6002: 49.50 ± 10.96 s; *F*_1,31_ = 2.99, *p* = 0.031; *n* = 7–9) and increased the time spent in total grooming of RRS rats (Stress: 69.50 ± 10.14 s, Stress+KW‐6002: 109.60 ± 17.34 s; *F*_1,27_ = 13.27, *p* = 0.029; *n* = 4–10; Figure 1F). It is also important to mention that in non‐stressed rats KW‐6002 induced a slight non‐significant (*p* > 0.05) decrease of the distance traveled, number of entries, time spent in the open arms of the elevated plus maze and of the time spent in sucrose grooming in the splash test (Figure 1B–D,F). The administration of KW‐6002 in RRS rats also increased significantly the density of glucocorticoid receptors in the frontal cortex (Stress: 48.91% ± 13.72%; Stress+KW‐6002: 111.50% ± 18.07%; *F*_1,34_ = 10.00, *p* = 0.017; *n* = 8–11), to levels similar to those of the control group (*F*_1,33_ = 9.91, *p* = 0.519, *n* = 8–11), as observed in Figure 1G. Overall, the gathered data show that A_2A_R blockade attenuated the anxiety‐like and self‐care behavior and the decrease in glucocorticoid receptors density upon RRS.

### 3.4 A_2A_R Blockade Modulates RRS‐Induced Alteration of AQP4 Density and Polarization

In gliosomes from frontal cortex, the selective blockade of A_2A_R using KW‐6002 in animals submitted to RRS prevented the stress‐induced decrease of AQP4 density (Stress: 74.11% ± 5.92%, Stress+KW‐6002: 106.20% ± 11.55%; *F*_1,38_ = 4.55, *p* = 0.026; *n* = 9–13; Figure 2B) and of α‐syntrophin density (Stress: 52.60% ± 12.68%, Stress+KW‐6002: 90.86% ± 13.34%; *F*_1,19_ = 12.55, *p* = 0.045, *n* = 5–6, Figure 2C). Noteworthy, the perivascular AQP4 polarization ratio, evaluated through regional proximity with blood vessels (Figure 3A–C), was increased by KW‐6002 administration in RRS animals (Stress: 1.78 ± 0.07 a.u., Stress+KW‐6002: 2.40 ± 0.19 a.u.; *F*_1,24_ = 7.63, *p* = 0.009; *n* = 6–7; Figure 3B–D) to levels similar to those of non‐stressed animals. Furthermore, A_2A_R blockade prevented the RRS‐induced decrease in both perivascular AQP4 area coverage (Stress: 2.17% ± 0.31%; Stress+KW‐6002: 3.33% ± 0.41%; *F*_1,24_ = 6.04, *p* = 0.039; *n* = 6–9; Figure 3A,E) and cellular AQP4 area coverage (Stress: 36.00% ± 3.90%, Stress+KW‐6002: 54.33% ± 4.18%; *F*_1,24_ = 11.03, *p* = 0.001; *n* = 6–9; Figure 3F), which consequently increased total AQP4 area coverage as compared with animals submitted to RRS and administered with vehicle (Stress: 38.00% ± 3.95%, Stress+KW‐6002: 58.00% ± 4.71%; *F*_1,23_ = 13.02, *p* = 0.002; *n* = 6–7; Figure 3G). Our results demonstrate that A_2A_R blockade prevented impairments in AQP4 density in astrocytic endfeet, particularly in perivascular regions, impacting AQP4 polarization, which is thought to affect AQP4 function, and also prevented RRS‐induced alterations in area coverage.

## 4 Discussion

The main conclusion of the present study is the ability of adenosine A_2A_ receptors (A_2A_R) blockade to prevent aquaporin‐4 (AQP4) mis‐localization upon repeated restraint stress (RRS). This extends to an in vivo setting the previous in vitro findings in cultured astrocytes showing that A_2A_R controlled AQP4 polarization upon prolonged exposure to glucocorticoids, mimicking stress‐like conditions (Dias et al. 2025). Since A_2A_R overfunction (Cunha et al. 2006; Kaster et al. 2015) and AQP4 dysfunction (Wei et al. 2019; Bollinger et al. 2025) are simultaneously observed in preclinical models of psychological stress, the present observation prompts the new working hypothesis that the ability of A_2A_R antagonist to prevent stress‐induced behavioral deficits (Kaster et al. 2015; Wang et al. 2023) might involve the control of astrocytic AQP4‐mediated processes (reviewed in Salman et al. 2021).

Previous studies have reported alterations of AQP4 in rodents subject to chronic stress (Wei et al. 2019; Wen et al. 2024; Bollinger et al. 2025) as well as in *post‐mortem* brain tissue from patients with major depressive disorder (Azis et al. 2019; Rajkowska et al. 2013; Gur et al. 2020). Importantly, stress and depression involve a loss of perivascular AQP4 polarization, as also shown in the present study, which is consistent with a potential dysfunction of the glymphatic system linked to mood deterioration (Gu et al. 2022; Wen et al. 2024). Accordingly, the genetic deletion of AQP4 exacerbates corticosterone‐induced depression (Kong et al. 2014) and AQP4 polymorphisms are associated with vascular depression (Westermair et al. 2018). Moreover, mice subject to chronic unpredictable stress display a dysfunction of AQP4 in perivascular astrocytes of the prefrontal cortex that are associated with behavioral deficits (Bollinger et al. 2025). AQP4 dysfunction has been proposed to causally link depression with sleep disturbance, which is a common concomitant and prodromal symptom of mood disorders (Yan et al. 2021). AQP4 dysfunction has also been identified as a link between depression and memory deterioration (Xia et al. 2017), and protecting the cerebrovascular and glymphatic systems alleviates depression‐incident cognitive dysfunction (Liu et al. 2020). Overall, the available evidence is indicative of a role of AQP4 dysfunction in the behavioral deterioration associated with depressive conditions (Genel et al. 2021). Therefore, the restoration of depression‐induced AQP4 mis‐localization may be a new strategy to alleviate the burden of depression, as hinted by the involvement of AQP4 in the effects of the antidepressant fluoxetine (Di Benedetto et al. 2016). Importantly, AQP4 redistribution has been increasingly recognized as a relevant pathological mechanism in other brain disorders (Alhadidi et al. 2024), including brain edema, where altered AQP4 expression and translocation contribute to disease progression (Kitchen et al. 2020). In fact, pharmacological strategies targeting AQP4 localization reduce edema and improve functional outcomes (Kitchen et al. 2020; Sylvain et al. 2021), supporting the therapeutic relevance of modulating AQP4 subcellular distribution. In this context, the RRS‐induced loss of perivascular AQP4 polarization observed in the present study further suggests that interventions aimed at preserving or restoring AQP4 localization might represent a promising target to mitigate perivascular astrocytic alterations associated with maladaptive stress.

A_2A_R is a candidate new target for antidepressants based on observation that the antagonism of A_2A_R prevents mood and memory dysfunction upon repeated stress (Cunha et al. 2006; Kaster et al. 2015; Wang et al. 2023) and A_2A_R polymorphisms are associated with depression (Oliveira et al. 2019). However, the mechanisms operated by A_2A_R to prevent brain damage are still unclear (reviewed in Cunha 2016). Previous studies argued for the involvement of neuronal processes (Wang et al. 2023), in particular the control of abnormal synaptic plasticity (Kaster et al. 2015). The present results posit the new hypothesis that A_2A_R‐mediated attenuation of anxiety‐like behavioral deficits may also involve a control of AQP4 mis‐localization, which might have a potential impact in restoring the function of the neurovascular unit and the glymphatic system. This concluded ability of A_2A_R to control AQP4 mis‐localization upon RRS joins previous observations in different models of brain disease showing that A_2A_R control AQP4 function in other noxious brain conditions such as TBI (Zhao et al. 2017) or sleep deprivation (Sun et al. 2024). Considering that RRS affects the hypothalamic–pituitary–adrenal (HPA) axis, leading to increased glucocorticoid (corticosterone) levels in serum (Chiba et al. 2012), and our previous in vitro findings showed that prolonged glucocorticoid exposure affects AQP4 localization (Dias et al. 2025), it is possible that HPA axis dysregulation contributes to the AQP4 mis‐localization observed in the present study. A previous studies showed that A_2A_R blockade normalized corticosterone oscillations under stress conditions (Batalha et al. 2013). Our data only show that A_2A_R blockade normalized total GR density without providing plasma corticosterone levels; this does not allow distinguishing transcriptional regulation from altered trafficking or phosphorylation of GR, which should be detailed in future studies by studying the impact of A_2A_R and stress on the nuclear versus cytosolic GR localization in the cortex. In parallel, previous studies also showed that A_2A_R blockade prevents stress‐induced impairments in long‐term potentiation (LTP), indicating restoration of synaptic function (Batalha et al. 2013; Kaster et al. 2015), and therefore we cannot fully exclude that improved behavior following KW‐6002 treatment may partially reflect normalization of neuronal network excitability. Additionally, since KW‐6002 was administered systemically, we cannot exclude peripheral contributions to the observed effects. It is important to emphasize that the timing of A_2A_R antagonism is critical in determining its effects, as these depend on whether it is administered before, during, or after disease onset (Chen et al. 2013; Franco and Navarro 2018). In the present study, KW‐6002 was administered before the onset and throughout the RRS protocol, suggesting a protective effect. Future studies should define if KW‐6002 might also be effective therapeutically to revert AQP4 mislocalization in pre‐installed mood diseases.

A_2A_R also exert context‐dependent effects under non‐pathologic and pathologic conditions: under homeostatic conditions, A_2A_R contribute to the fine regulation of neuronal activity and signaling balance (Chen et al. 2013; Cunha 2016); however, stressful conditions are associated with increased extracellular adenosine levels and A_2A_R upregulation, potentially shifting this balance toward maladaptive signaling (Cunha et al. 2006; Batalha et al. 2013; Kaster et al. 2015). Thus, although A_2A_R blockade affords both prophylactic and therapeutic protective effects in models of chronic stress (Kaster et al. 2015) as well as in other brain diseases (Canas et al. 2009; Orr et al. 2015; Nunes et al. 2024), stress‐induced A_2A_R upregulation might affect AQP4 localization through a different transducing system compared to control conditions, as previously reported in other brain disorder conditions (e.g., Dai et al. 2010). Although we did not explore the mechanisms underlying the A_2A_R‐AQP4 interaction, we hypothesize an involvement of protein kinase A (PKA) or protein kinase C (PKC), which are activated downstream of A_2A_R signaling and are known to be involved in the motility of AQP4‐containing vesicles, AQP4 internalization into vesicles and translocation from plasma membrane to intracellular compartments, regulating its cell surface density and water permeability (Carmosino et al. 2007; Fenton et al. 2010; Potokar et al. 2013; Kitchen et al. 2015, 2020; Noël et al. 2021). Our findings provide evidence of a redistribution of AQP4, consistent with impaired perivascular astrocytic organization under stress conditions, which anticipate modification of the glymphatic system, although functional assessment of CSF‐ISF exchange will be further required to directly establish glymphatic transport deficits in this model. Importantly, the ability of A_2A_R to control AQP4 localization and activity fills a current critical need of effective modulators to control the activity of AQP4 (Papadopoulos and Verkman 2013; Salman et al. 2021). Future studies should aim to demonstrate the ability of tinkering with A_2A_R activity to restore the activity of the glymphatic upon stress‐induced mood dysfunction.

## Author Contributions

**Ana Margarida Nabais:** methodology, investigation. **Rodrigo A. Cunha:** writing – review and editing, validation, funding acquisition, supervision. **Samira G. Ferreira:** methodology, formal analysis, investigation. **Paula Agostinho:** conceptualization, methodology, supervision, project administration, writing – review and editing, resources, validation. **Joana Silva:** methodology, investigation. **Liliana Dias:** data curation, formal analysis, writing – original draft, visualization, methodology, investigation.

## Funding

This work was supported by the Centro 2030 (CENTRO2030‐FEDER‐02359100), FCT (COMPETE2030‐FEDER‐0067630 and UIDB/04539/2020) and European Union's Horizon 2020 research and innovation program (grant agreement No 857524). L.D. was supported by a fellowship from FCT—*Fundação para a Ciência e Tecnologia* (SFRH/BD/147159/2019).

## Ethics Statement

Animals were handled according to the principles and procedures outlined as “3R's” in the FELASA, ARRIVE, and European Union guidelines (86/609/EEC) and were approved by the Animal Welfare Committee of the CNC (ORBEA_238_2019/1410/2019).

## Conflicts of Interest

The authors declare no conflicts of interest.

## Supporting information


**Figure S1:** Repeated restraint stress in male Wistar rats did not affect GR or AQP4 density in hippocampus. In the hippocampus, restraint stress (Stress) did not affect glucocorticoid receptors (GR) density (A), total aquaporin‐4 (AQP4) density (B) nor AQP4 density in gliosomes (corresponding to astrocytic membrane endfeet), evaluated by Western blot (C). Representative images (rearranged for presentation purposes) of GR, AQP4 and β‐actin (loading control protein) immunoblots are shown below the respective panel. Data are mean ± SEM of 6–9 rats per group; *p* > 0.05 as compared with non‐stressed animals (control, CTRL) using a one‐way ANOVA followed by Dunnett's *post hoc* test.

## Data Availability

The data that support the findings of this study are available from the corresponding author upon reasonable request.
