# Supplementary material for: Adenosine A2A Receptors Control the Mis‐Localization of Aquaporin‐4 in Rats Subject to Repeated Restraint Stress
Source: J Neurochem. 2026 Apr 27;170:e70450. doi: 10.1111/jnc.70450 (PMC13116037; doi:10.1111/jnc.70450)
Supplement: Supplementary file 1 — Figure S1: Repeated restraint stress in male Wistar rats did not affect GR or AQP4 density in hippocampus. In the hippocampus, restraint stress (Stress) did not affect glucocorticoid receptors (GR) density (A), total aquaporin‐4 (AQP4) density (B) nor AQP4 density in gliosomes (corresponding to astrocytic membrane endfeet), evaluated by Western blot (C). Representative images (rearranged for presentation purposes) of GR, AQP4 and β‐actin (loading control protein) immunoblots are shown below the respective panel. Data are mean ± SEM of 6–9 rats per group; p > 0.05 as compared with non‐stressed animals (control, CTRL) using a one‐way ANOVA followed by Dunnett's post hoc test. [file JNC-170-e70450-s001.docx]

**Supplementary Data**

**ADENOSINE A_2A_ RECEPTORS CONTROL THE MIS-LOCALIZATION OF AQUAPORIN-4 IN RATS SUBJECT TO REPEATED RESTRAINT STRESS**

Liliana Dias^1^, Samira G. Ferreira^1,2^; Ana Margarida Nabais^1^, Joana Silva^1^, Rodrigo A. Cunha^1,3,4^, Paula Agostinho^1,3^

^1^CiBB - Centre for Innovative Biomedicine and Biotechnology, ^2^Department of Life Sciences, Faculty of Sciences and Technology, ^3^Faculty of Medicine, ^4^MIA-Portugal, Multidisciplinary Institute of Aging, University of Coimbra, Portugal

**
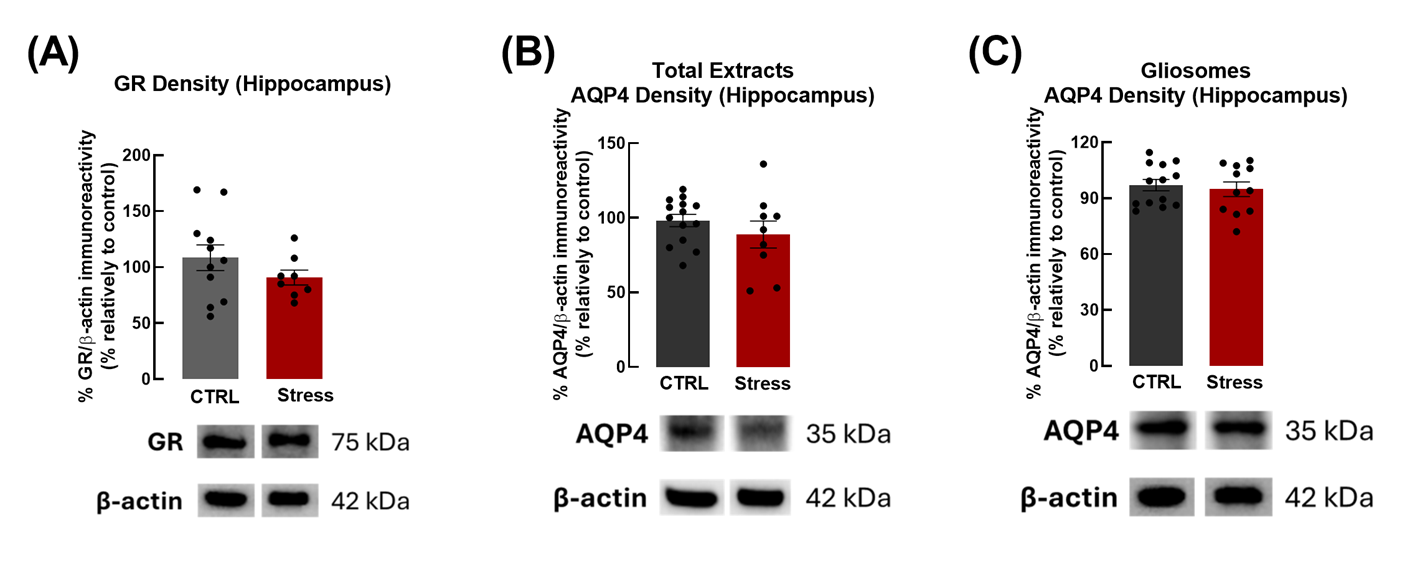
**

**Fig.S1 –** **Repeated restraint stress in male Wistar rats did not affect GR or AQP4 density in hippocampus.** In the hippocampus, restraint stress (Stress) did not affect glucocorticoid receptors (GR) density **(A)**, total aquaporin-4 (AQP4) density **(B)** nor AQP4 density in gliosomes (corresponding to astrocytic membrane endfeet), evaluated by Western blot **(C)**. Representative images (rearranged for presentation purposes) of GR, AQP4 and β-actin (loading control protein) immunoblots are shown below the respective panel. Data are mean ± SEM of 6-9 rats per group; p>0.05 as compared with non-stressed animals (control, CTRL) using a one-way ANOVA followed by Dunnett’s *post hoc* test
